# Redox destabilization by ibrutinib promotes ferroptosis in diffuse large B-cell lymphoma (DLBCL)

**DOI:** 10.1038/s41420-025-02826-w

**Published:** 2025-10-31

**Authors:** Anuschka Langpape, Debora Bonasera, Jenny Stroh, Moritz Reese, Maria Cartolano, Gianmaria Liccardi, Silvia von Karstedt

**Affiliations:** 1 Department of Translational Genomics, University of Cologne, Faculty of Medicine and University Hospital Cologne, Cologne, Germany; 2 CECAD Cluster of Excellence, Faculty of Medicine and University Hospital Cologne, Cologne, Germany; 3 Genome instability, inflammation and cell death laboratory, Institute of Biochemistry I, Centre for Biochemistry, Faculty of Medicine, University of Cologne, Cologne, Germany; 4 Cell death, inflammation and immunity laboratory, Institute of Biochemistry I, Centre for Biochemistry, Faculty of Medicine, University of Cologne, Cologne, Germany; 5 Cell death, inflammation and immunity laboratory, CECAD Cluster of Excellence, University of Cologne, Cologne, Germany; 6 Center for Molecular Medicine Cologne, Faculty of Medicine and University Hospital Cologne, Cologne, Germany

**Keywords:** Cell death, Haematological cancer

## Abstract

Diffuse large B-cell lymphoma (DLBCL) exhibits marked clinical heterogeneity and frequent treatment resistance, particularly in molecularly defined high-risk subtypes such as ABC-DLBCL. While current therapies largely rely on apoptosis induction, non-apoptotic cell death pathways remain underexplored in hematologic malignancies. Here, we identify ferroptosis, an iron-dependent, lipid peroxidation-driven form of regulated necrosis, as an effective baseline in additive therapy with ibrutinib for the treatment of DLBCL. Transcriptomic and lipidomic analyses revealed that DLBCL cells, despite lacking overt enrichment of polyunsaturated fatty acids (PUFAs), display elevated expression of the core ferroptosis protective machinery. Inhibition of GPX4 induced rapid and selective lipid ROS accumulation and cell death across a panel of human and murine DLBCL cellular models irrespective of subtype. Notably, the BTK inhibitor and clinical compound ibrutinib showed additive effects with GPX4 inhibition, even at concentrations below its cytotoxic threshold, expanding its therapeutic relevance beyond BTK inhibition. Mechanistically, we uncover two activities of ibrutinib to enhance ferroptosis sensitivity: First, chemical scavenging of glutathione and second the inhibition of GPX4 protein expression via translational repression. Thereby, our findings define ferroptosis as a basis for additive therapy in combination with ibrutinib in DLBCL and reveal a previously unrecognized role for ibrutinib in directly modulating anti-oxidant defense.

## Introduction

Diffuse large B-cell lymphoma (DLBCL) is the most common type of aggressive non-Hodgkin lymphoma, in which current standard-of-care achieves cure rates in approximately 60% of patients [1]. Yet a substantial proportion of patients exhibit primary resistance or relapse following partial remission. This heterogeneous response pattern is likely owed to the fact that the overarching DLBCL entity comprises various molecular subtypes with very distinct individual biology and, hence, response and prognosis. DLBCL has been classified into germinal center B-cell–like (GCB) and activated B-cell–like (ABC) subtypes based on gene expression profiling [2]. Notably, ABC-DLBCL is associated with inferior outcomes and reduced responsiveness to frontline regimens [3]. These observations highlight an urgent need to define and exploit novel molecular vulnerabilities in high-risk subtypes, particularly ABC-DLBCL. Molecularly, ABC-DLBCL frequently harbors co-occurring mutations in MYD88, CD79A/B, CDKN2A, and TNFAIP3, which converge on chronic NF-κB pathway activation [4]. Furthermore, NF-κB signaling can be amplified through formation of the My-T-BCR supercomplex, comprising TLR9 and the B-cell receptor (BCR) within endolysosomes [5]. This complex drives tonic NF-κB signaling and cytokine production, including IL-6 and IL-10 [6]. In contrast, GCB-DLBCL more commonly displays alterations in EZH2, CREBBP, SGK1, and BCL2 translocations [4, 7]. These alterations collectively lead to persistent expression of anti-apoptotic and pro-survival genes thereby contributing to apoptosis resistance. Although BH3 mimetics and BTK inhibitors in combination with Rituximab, Cyclophosphamide, Hydroxydaunorubicin, Oncovin, and Prednisone (R-CHOP) have shown additional benefit [8, 9], this does not hold true for all patients and relapse remains a problem, especially in ABC-DLBCL and other high-risk molecular subtypes.

Beyond resistance to canonical apoptosis, emerging evidence suggests that drug-tolerant persister cells acquire selective mesenchymal states dependent upon lipid-directed anti-oxidant defense [10, 11]. One such pathway is ferroptosis, an iron-dependent form of regulated necrosis. Molecularly, ferroptosis is characterized by a chain reaction of toxic membrane lipid peroxidation [12] triggered upon deletion of glutathione peroxidase 4 (GPX4) [13, 14], its pharmacological inhibition or through limiting availability of its co-factor glutathione (GSH) [15]. In addition to this GSH-dependent route of ferroptosis protection, the COQ10 oxidoreductase ferroptosis suppressor protein 1 (FSP1, formerly AIFM2) protects cancer cell lines of various tissue origins from ferroptosis through the generation of the lipid radical-trapping agent ubiquinole [16, 17]. During ferroptosis, specific phosphatidylethanolamine (PE) lipids, including polyunsaturated fatty acids (PUFAs) such as arachidonic acid species, are peroxidized [18]. This results in an excessive lipid peroxidation profile observed in ferroptosis but not other types of regulated cell death [19]. Herein, presence of redox active iron, which can be imported through CD71, with H2O2 gives rise to a Fenton reaction, which generates hydroxyl radicals. These are highly reactive reactive oxygen species (ROS) which ultimately trigger lipid peroxidation in a chain reaction driven by lipid ROS [15]. Synthesis of these lipids is catalyzed by Acyl-CoA Synthetase Long Chain Family Member 4 (ACSL4), whose expression is therefore a prerequisite for cellular ferroptosis sensitivity [20]. Importantly, cancer cells with high oxidative stress or altered redox homeostasis, such as DLBCL, appear particularly vulnerable to ferroptosis [21]. In fact, a screen across 100 cancer cell lines identified DLBCL as the most ferroptosis-sensitive tumor type [13], highlighting a potentially actionable vulnerability. Indeed, GPX4 inhibition or depletion of GSH can induce ferroptosis in DLBCL cells [13, 22]. Notably, glutaminase 1 (GLS1) inhibition was shown to trigger oxidative stress and ferroptotic cell death in DLBCL, an effect enhanced by co-treatment with venetoclax [23]. Similarly, in GCB-DLBCL, elevated expression of T-Complex 1 (TCP1) correlated with increased sensitivity to GPX4 small molecule inhibitors such as RSL3 [24]. Despite these promising leads, ferroptosis-inducing agents alone often require high doses, which may be limited by toxicity. Therefore, rational combination strategies are needed to potentiate ferroptosis in cells with particular dependence while minimizing off-target effects.

Here, we explore the potential of Bruton’s tyrosine kinase (BTK) inhibition to sensitize DLBCL cells to ferroptotic death. We show that ibrutinib not only exhibits additive activity when combined with GPX4 inhibitors but also enhances ferroptosis sensitivity by reducing intracellular glutathione and downregulating GPX4 protein expression. Co-targeting BTK and GPX4 achieves robust ferroptotic killing in DLBCL cellular models irrespective of subtype. These findings establish a mechanistic framework for integrating ferroptosis-based strategies into the treatment of aggressive, treatment-refractory DLBCL.

## Results

### DLBCL exhibits a phenotype suggestive of active lipid antioxidant defense

**Fig. 1:**
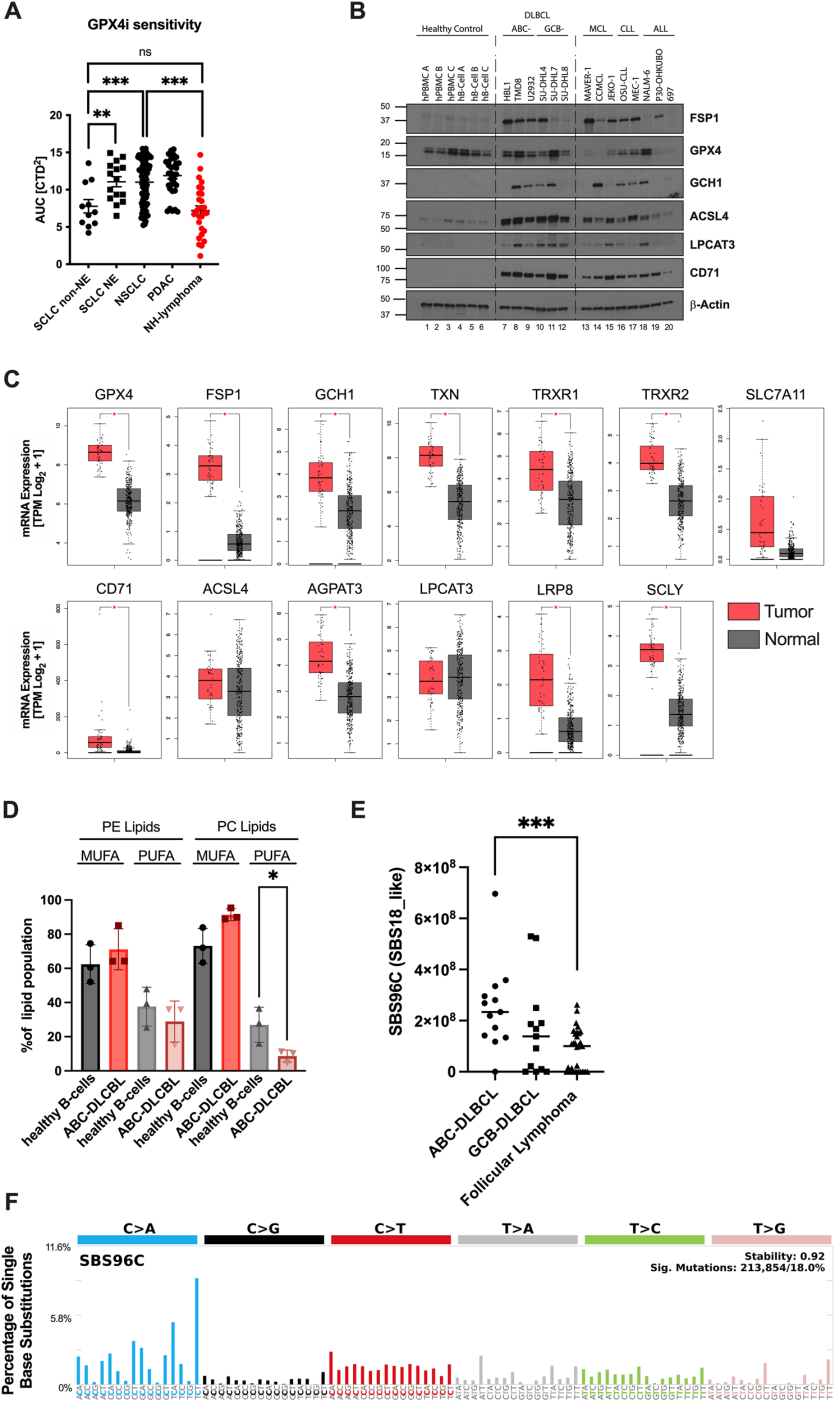
DLBCL exhibits a phenotype suggestive of active lipid antioxidant defense. **A** ML210-treated human SCLC non-NE (*n* = 11), SCLC NE (*n* = 15), NSCLC (*n* = 100), PDAC (*n* = 34), and NH-lymphoma (*n* = 27) cells were plotted for area under the curve (AUC). Source data was obtained from Depmap (DepMap, Broad, 2024). **B** Representative western blot of ferroptosis pathway component expression in tumor cells of B-cell malignancies. **C** mRNA expression of the indicated gene in the TCGA DLBCL (*n* = 47) cohort was compared to GTEX normal B cells (*n* = 337). Data analysis was performed using http://gepia.cancer-pku.cn/index.html. T tumour, N normal. **D** ABC-DLBCL cells (HBL1, TMD8, U2932) and human B-cells from three healthy donors were analyzed by lipidomics. Percent of total lipid populations per analysis group was plotted for phosphatidylethanolamine (PE) and phosphatidylcholines (PC) species. **E** SBS96C signature abundance in DLBCL and follicular lymphoma. **F** Composition of the SBS96C signature. Data are mean ± SD, statistics tested by unpaired *t*-test (**A**, **D**, **E**); *= *p* value 0.05, **= *p* value 0.01, ***= *p* value 0.001.

Ferroptosis has emerged as potential vulnerability in various cancers [25–28]. To benchmark ferroptosis sensitivity of non-Hodgkin lymphomas (NHL) in direct comparison to other cancer entities with known ferroptosis sensitivity and resistance, we mined publicly available datasets for cancer entity cell line panels using DepMap [29]. As a control, we could independently confirm that non-neuroendocrine (non-NE) small cell lung cancer (SCLC) presents with a relatively small area under the curve (AUC) and, hence, is highly ferroptosis sensitive in response to the GPX4 small molecule inhibitor ML210 [30]. Moreover, the dataset equally confirmed recently published data demonstrating that GPX4 targeting is inefficient in pancreatic cancer [31]. Interestingly, a group within NHL cell lines was highly sensitive to ferroptosis in comparison to the validated ferroptosis resistant (NE SCLC, NSCLC, PDAC) and sensitive (non-NE SCLC) controls (Fig. 1A). Yet, the data distribution within NHL suggested potential subtype heterogeneity regarding ferroptosis sensitivity.

Therefore, we next assessed protein expression of ferroptosis regulators across different human B-cell–derived hematologic malignancies in comparison to normal human peripheral blood mononuclear cells (PBMCs) and purified normal human B-cells. Interestingly, while normal cells readily expressed GPX4, additional fail-safe mechanisms protecting from ferroptosis (FSP1, GCH1) were upregulated selectively in malignant cells suggesting dependence on ferroptosis protection (Fig. 1B). Notably, both ABC- and GCB-type DLBCL cells showed strong expression of ACSL4 and CD71, suggesting the presence of a ferroptosis-amenable lipidome and intracellular availability of redox active iron to be present in DLBCL. Similarly, cells derived from a murine DLBCL lymphoma model [32] also exhibited elevated expression of CD71 and the core ferroptosis molecular machinery in comparison to normal murine B-cells (Suppl. Fig. 1A). To next determine whether clinical samples from DLBCL patients would reflect this protein expression profile at the transcriptomic level, we analyzed mRNA expression of canonical ferroptosis-related genes in comparison to normal B-cells using the DLBCL cohort (DLBC) within The Cancer Genome Atlas (TCGA) as well as the normal B-cell cohort from the Genotype-Tissue Expression (GTEx) Portal. Indeed, across a cohort of 47 DLBCL tumor samples, CD71, GPX4, FSP1, ACSL4, TXN, TRXR1, TRXR2, LRP8, SCLY, and AGPAT3 transcripts were all significantly elevated compared to normal B-cell controls (Fig. 1C). This transcriptional program suggested a metabolic investment of DLBCL cells in lipid-associated anti-oxidant defense. Because ferroptosis execution relies on the presence of oxidizable phospholipids, particularly polyunsaturated fatty acid–containing phosphatidylethanolamines (PUFA-PEs), we therefore next profiled the lipid composition of three ABC-DLBCL cell lines in comparison to primary human B-cells from healthy donors. Interestingly, the PUFA content was downregulated in DLBCL cells (Fig. 1D and Suppl. Fig. 1B, C). Moreover, ABC-DLBCL lines displayed a relative enrichment of monounsaturated and saturated fatty acids (MUFA/SFA) at the expense of PUFA species. These findings suggested that DLBCL cells might also have adapted PUFA content in response to constitutive high levels of ROS. Indeed, when analysing somatic mutational signatures in ABC- and GCB-DLBCL versus follicular lymphoma (FL), we found higher presence of a ROS-associated mutational signature in ABC-DLBCL patients in particular (Fig. 1E, F).

Taken together, these results uncover that DLBCL presents with a protein, RNA, and lipidome phenotype suggestive of elevated endogenous lipid-directed oxidative stress.

### DLBCL shows superior sensitivity to GPX4 inhibition over normal B-cells

**Fig. 2:**
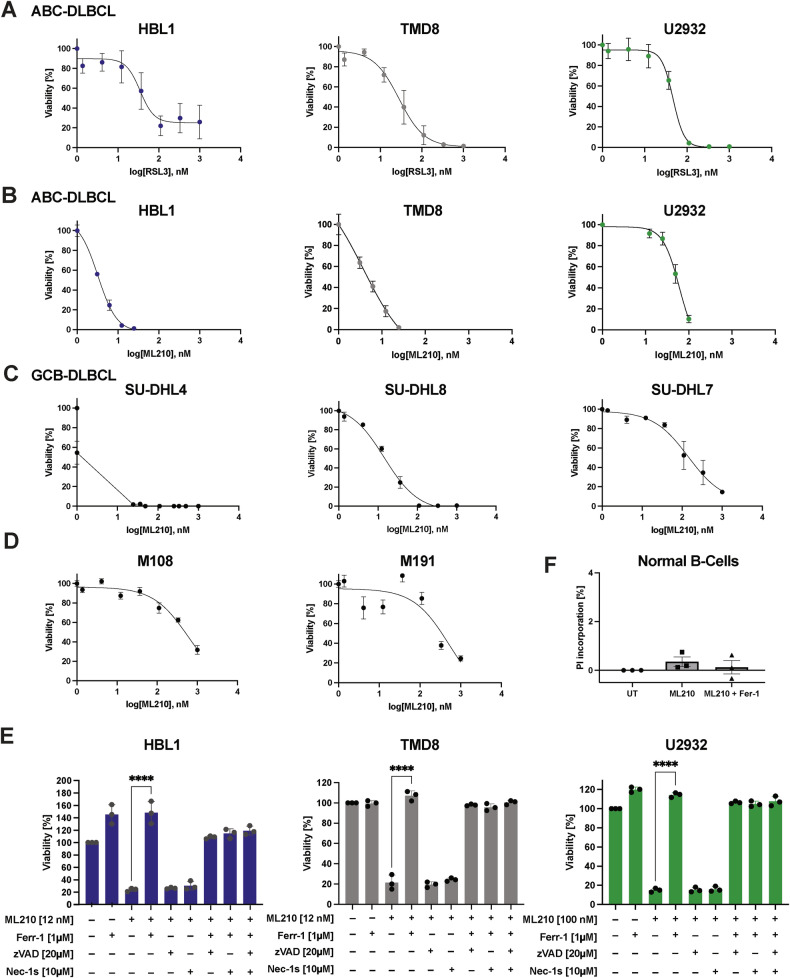
DLBCL shows superior sensitivity to GPX4 inhibition over normal B cells. Three human ABC-DLBCL cell lines were treated with **A** RSL3 and **B** ML210 with the indicated concentrations of the respective drug. Cell viability was determined by CellTiter-Glo® after 48 h. **C** The indicated human GCB-DLBCL cell lines were treated with increasing ML210 concentrations. Cell viability was determined by CellTiter-Glo® after 48 h. **D** Murine DLBCL cell lines were treated with the indicated concentrations of ML210. **E** Rescue experiments of human ABC-DLBCL cell lines treated with indicated concentrations of ML210, Fer-1, zVAD, and Nec-1s for 48 h. Cell viability was measured by CellTiter-Glo®. **F** Propidium iodide (PI) incorporation measured by flow cytometry after 48 h of treating B-cells isolated from PBMCs of three healthy donors with ML210 [1 µM] and Fer-1 [1 µM]. Data are mean ± SD for each cell line of *n* = 3 independent experiments. one-way ANOVA. *= *p* value 0.05, **= *p* value 0.01, ***= *p* value 0.001, ****= *p* value 0.0001.

Having established that DLBCL cells show signs of endogenous lipid-directed oxidative stress, we next evaluated whether this would translate into vulnerability to ferroptotic cell death. Despite the discovery of a role for FSP1 in protecting from ferroptosis [16, 17], its small molecule targeting is mostly insufficient to induce ferroptosis in cells expressing GPX4. Given that GPX4 is therefore still the major pharmacological target to trigger ferroptosis using small molecule inhibitors, we treated a panel of human ABC-DLBCL (U2932, HBL1, TMD8) and GCB-DLBCL (SU-DHL4, SU-DHL7, SU-DHL8) cell lines with two chemically distinct GPX4 inhibitors, ML210 and RSL3. All DLBCL lines demonstrated a steep, dose-dependent decline in viability within the low nanomolar to sub-micromolar range following GPX4 inhibition, with no consistent difference in sensitivity between ABC and GCB subtypes (Fig. 2A–C). Moreover, this efficacy extended to cell lines derived from a mouse model developing lymphoma as a result of oncogenic B-cell specific expression of Myd88^L252P^ and BCL2 [32], which mimic the constitutive NF-κB activation observed in ABC-type DLBCL (Fig. 2D). Notably, DLBCL is commonly treated with therapeutic regimens such as R-CHOP, which includes DNA-crosslinking agents comparable to cisplatin. While ABC-DLBCL cell lines showed dose-dependent sensitivity to cisplatin, with approximately 50% viability loss occurring at concentrations around 10 µM (Suppl. Fig. 2), the same cells underwent near-complete ferroptotic death at ML210 concentrations below 1 µM. These data suggested that GPX4 inhibition is highly efficacious across human and murine DLBCL. Mechanistically, ferroptosis is a regulated, iron-dependent form of necrosis distinct from apoptosis or necroptosis. To verify the nature of viability loss induced by GPX4 inhibition, we conducted rescue experiments in ABC-DLBCL lines using ferrostatin-1 (Fer-1), a lipophilic antioxidant that suppresses ferroptosis, alongside zVAD-fmk (a pan-caspase inhibitor) and necrostatin-1s (Nec-1s, a RIPK1 inhibitor). Importantly, only Fer-1 significantly restored viability in ML210- and RSL3-treated cells, confirming that cell death was ferroptotic rather than apoptotic or necroptotic (Fig. 2E and Suppl. Fig. 3). In contrast, zVAD-fmk and Nec-1s had no protective effect, consistent with a non-canonical, caspase-independent death mechanism. Furthermore, to evaluate the therapeutic selectivity of ferroptosis induction, we examined whether GPX4 inhibition impacts healthy B-cells. CD19^+^ B-cells isolated from three healthy donors were treated with high doses of ML210 and quantified for cell death induction (PI incorporation) by flow cytometry (Fig. 2F and Suppl. Fig. 4). Importantly, no significant increase in cell death was observed, with or without Fer-1 co-treatment, indicating that non-malignant B-cells are largely resistant to ferroptosis induction at therapeutic concentrations. This selective vulnerability of malignant over healthy B-cells is in stark contrast to cisplatin, which lacks tumor specificity and is known to damage normal tissues, including hematopoietic and immune compartments, during DLBCL treatment. Collectively, these results demonstrate that DLBCL cells, regardless of subtype, exhibit profound sensitivity to ferroptosis induction via small molecule GPX4 inhibition, and that this vulnerability is specific to malignant B-cells, supporting ferroptosis as a selective and mechanistically distinct strategy for therapeutic intervention in aggressive lymphomas.

### Ibrutinib and GPX4 inhibition show additive induction of lipid ROS and cell death in DLBCL cells

**Fig. 3:**
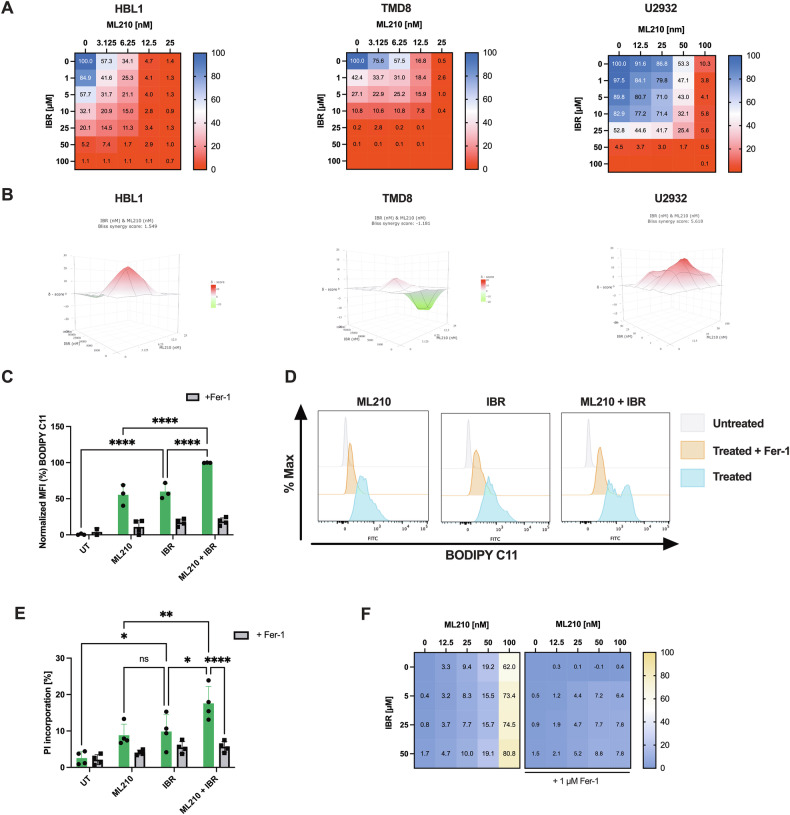
Ibrutinib and GPX4 inhibition show additive induction of lipid ROS and cell death in DLBCL cells. **A**, **B** The indicated human ABC-DLBCL cell lines were subjected to crosstitrations with increasing concentrations of ML210 and ibrutinib (IBR) for 48 h and cell viability was determined by CellTiter-Glo®. Heatmap color code indicates viability levels of each sample. BLISS synergy scores were determined using https://synergyfinder.fimm.fi. **C** U2932 cells were treated with IBR [25 µM], ML210 [50 nM] in the presence or absence of Fer-1 [1 µM] for 2.5 h followed by Lipid ROS readout using BODIPY C11 staining [5 µM] via flow cytometry. **D** Representative histogram of BODIPY C11 staining in (**C**). **E** PI incorporation in U2932 cells measured by flow cytometry after 2.5 h treatment with IBR [25 µM], ML210 [50 nM] in the presence or absence of Fer-1 [1 µM]. **F** U2932 cells were treated with increasing concentrations of IBR and ML210 in the presence or absence of Fer-1 [µM] and cell death was measured after 48 h via PI incorporation using flow cytometry. Data are mean ± SD of *n* = 3, biologically independent replicates. 2-way ANOVA. *= *p* value 0.05, **= *p* value 0.01, ***= *p* value 0.001, ****= *p* value 0.0001.

Given the frequent presence of activating MYD88 mutations in ABC-DLBCL and a resulting overactivation of the NF-κB pathway, Bruton’s tyrosine kinase (BTK) inhibitors -which can block downstream NF-κB- have been used in second line treatment of ABC-DLBCL [33–35]. Yet, also here, frequent development of resistance is observed causing a worse prognosis of the ABC-DLBCL a subtype. Therefore, we investigated whether ibrutinib might be combined with ferroptosis induction in DLBCL. To this end, we treated a panel of ABC-DLBCL cell lines (U2932, TMD8, and HBL1) with increasing concentrations of the GPX4 inhibitor ML210, in the presence or absence of rising concentrations of ibrutinib. Importantly, in all three models, ibrutinib substantially enhanced ML210-induced cytotoxicity (Fig. 3A). To further characterize the type of interaction observed, we applied BLISS independence modeling, which identified an additive interaction between ibrutinib and ML210 across the tested dose ranges (Fig. 3B). Similar effects were observed in GCB-DLBCL cell lines (Suppl. Fig. 5A, B). These findings were further validated using RSL3, an independent and structurally distinct GPX4 inhibitor. In both U2932 and TMD8 cells, RSL3 combined with ibrutinib similarly reduced cell viability in an additive effect (Suppl. Fig. 5C, D), underscoring the robustness of this combination. Notably, an observable hallmark of ferroptosis is the induction of lipid ROS which can be traced by BODIPY C11 oxidation [12]. While ML210 as well as ibrutinib alone both induced significant levels, combined treatment showed an additive effect of BODIPY C11 oxidation and, hence, lipid ROS induction, which was reverted by co-treatment with Fer-1 (Fig. 3C, D). Along with additive induction of lipid ROS, we also observed additive induction of cell death, which was neutralized by co-treatment with Fer-1 (Fig. 3E). Together, these data establish that ibrutinib shows additive activity with GPX4 inhibition to trigger lipid ROS-dependent ferroptotic cell death in ABC-DLBCL. This activity is reproducible across independent GPX4 inhibitors and selective towards the ferroptotic pathway.

### Ibrutinib depletes glutathione and GPX4

**Fig. 4:**
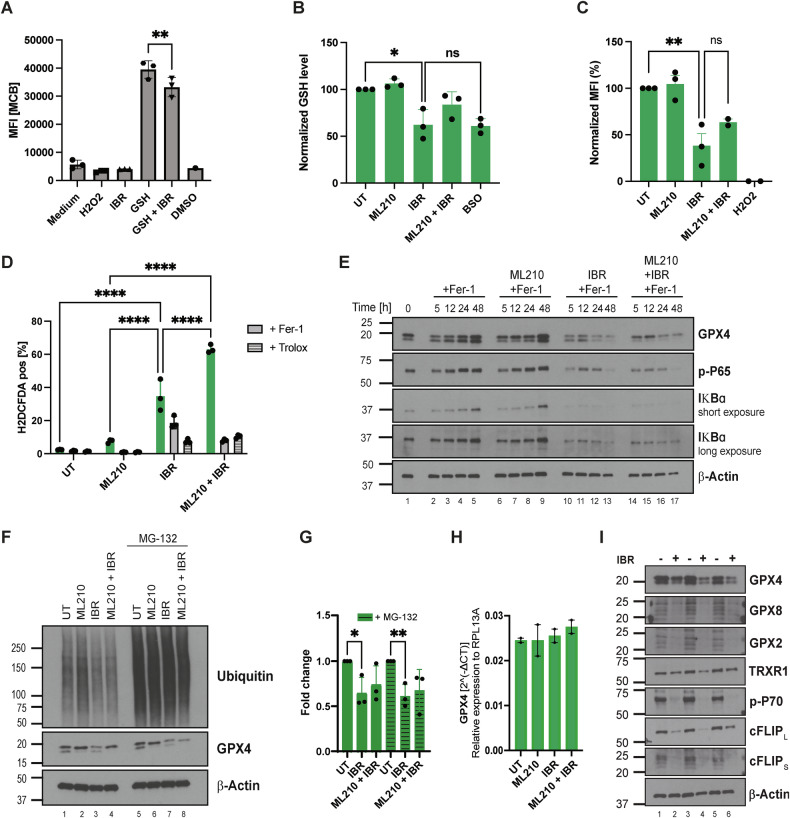
Ibrutinib depletes glutathione and GPX4. **A** Medium fluorescent intensity of MCB in a cell-free system with added Ibrutinib [25 µM], GSH [50 µM], H_2_O_2_ [100 µM], and DMSO after 2 h of incubation and fluorescence read out [Ex/Em: 394/490 nm]. **B** GSH levels in U2932 cells were measured using the GSH Promega Kit after 2 h of treatment with ML210 [50 nM], IBR [25 µM], BSO [300 mM], and normalized to the untreated condition. **C** MCB assay measured via fluorescence readout [Ex/Em: 394/490 nm] of U2932 cells treated with IBR [25 µM], ML210 [50 nM], H_2_O_2_ [10 µM] for 2 h. **D** General ROS readout by H2DCFDA assay of U2932 cells after 2 h treatment with IBR [25 µM], ML210 [50 nM] in the presence or absence of Fer-1 [1 µM] or Trolox [50 µM]. **E** Representative western blot of U2932 cells treated with IBR [25 µM] and ML210 [50 nM] in the presence of Fer-1 [1 µM]. **F** Representative western blot of U2932 cells treated with IBR [25 µM], ML210 [50 nM] in the presence of Fer-1 [1 µM] for 24 h. MG-132 [10 µM] was added for the last 4 h of treatment. **G** Densitometry quantification of GPX4 protein levels in (**F**) and two additional biological repeats. **H** GPX4 transcript levels measured after treating U2932 cells with IBR [25 µM], ML210 [50 nM] in the presence of Fer-1 [1 µM] for 48 h. Transcript levels are normalized to untreated RPL13A levels after 48 h. **I** Western blot showing three independent replicates of U2932 cells untreated or treated with IBR [25 µM] in the presence of Fer-1 [1 µM] for 24 h. Data are mean ± SD of *n* = 3 (**A**–**D**, **G**), *n* = 2 (**H**); biologically independent replicates. Statistics done by paired *t*-test (**A**), unpaired *t*-test (**B**, **C**, **G**); 2-way ANOVA (**D**). *= *p* value 0.05, **= *p* value 0.01, ***= *p* value 0.001, ****= *p* value 0.0001.

The additive effect observed for lipid ROS induction suggested a functional cooperation between ibrutinib and GPX4 inhibition, a mechanism which remained to be explored. While ibrutinib is a well-established BTK inhibitor that disrupts downstream NF-κB signaling, a critical oncogenic node in ABC-DLBCL, our data revealed that the enhanced cell death induced by ibrutinib and GPX4 inhibition could be fully rescued by ferrostatin-1. This observation and the fact that ibrutinib targets reactive thiol groups within BTK prompted us to investigate whether ibrutinib itself might directly scavenge GSH, a tripetide with various reactive thiols. To this end, we measured the extent to which ibrutinib could quench fluorescence of a thiol-reactive fluorogenic tracer dye (monoclorobimnane, MCB), commonly used to quantify GSH levels, in a cell-free system. Indeed, while added GSH readily induced a fluorescent signal, pre-incubation of ibrutinib with GSH in a cell-free environment led to a significant decrease in fluorescent signal (Fig. 4A). Similarly, the same effect was observed in DLBCL cells with endogenous levels of GSH and the extent of GSH depletion was comparable to GSH depletion as a result of GSH synthesis inhibition using the γ-glutamylcysteine synthetase (γ-GCS) inhibitor buthionine sulphoximine (BSO) (Fig. 4B, C). Given the observed GSH depletion, we next examined whether ibrutinib would promote ferroptosis-relevant oxidative stress. While treatment with ML210 did not induce significant levels of cytosolic ROS, ibrutinib treatment and its combination with ML210 led to their significant induction (Fig. 4D). This increase was reversed by co-treatment with ferrostatin-1 or the antioxidant Trolox, suggesting ROS accumulation to be in part lipid ROS-dependent. These data suggested that one mechanism by which ibrutinib provided additivity was through its scavenging of GSH and, consequently, increased ROS feeding into lipid ROS while impairing GPX4 activity through limited availability of GSH. Interestingly, we noted a consistent decrease in GPX4 protein levels across multiple ABC- and GCB-DLBCL cell lines following ibrutinib exposure (Fig. 4E, Suppl. Fig. 6A), which was not reversible by addition of GSH (Suppl. Fig. 6B). Notably, the significant downregulation of GPX4 protein levels following ibrutinib treatment was not reversed by proteasome inhibition using MG-132 (Fig. 4F, G), indicating a proteasome-independent mechanism for the decreased protein level observed. To determine whether this regulation occurred at the transcriptional level, we performed quantitative RT-PCR. Yet, GPX4 transcript levels remained unchanged following ibrutinib treatment, indicating that the observed reduction in protein levels is not a result of impaired GPX4 transcription (Fig. 4H). Therefore, we next investigated whether ibrutinib interferes with translation. Strikingly, ibrutinib treatment led to a broad suppression of short-lived protein levels such as cFLIP, MCL-1, and cMYC, consistent with a more global impairment of protein translation (Fig. 4I and Suppl. Fig. 7A). These findings suggest that ibrutinib-mediated GPX4 loss is likely a consequence of translational repression rather than targeted degradation or transcriptional silencing.

Together, these data propose that ibrutinib affects lipid-ROS driven anti-oxidant defense at multiple levels (i) by depleting GSH, (ii) by suppressing GPX4 protein expression via translational inhibition, and as a result (iii) by amplifying lipid ROS. This multifaceted disruption of redox homeostasis significantly enhances DLBCL cell vulnerability to ferroptosis and provides a mechanistic rationale for exploiting BTK inhibitors in combination with ferroptosis-inducing therapies in high-risk, apoptosis-refractory lymphomas.

## Discussion

Despite advances in molecular subclassification and targeted therapy, treatment-resistant DLBCL, particularly the ABC subtype, remains a clinical challenge [1]. Standard-of-care regimens such as R-CHOP fail to induce durable remission in a significant proportion of patients, often due to apoptosis resistance and reliance on pro-survival signaling pathways, including constitutive NF-κB activation [36, 37]. While BTK inhibitors have shown efficacy in subsets of ABC-DLBCL [38], their activity as single agents is frequently limited by the emergence of adaptive resistance and incomplete suppression of tumor survival programs.

Besides previous studies highlighting ferroptosis vulnerability of DLBCL [13, 22, 23, 39], our study reveals that induction of ferroptosis can be used as a baseline for additive therapy using ibrutinib. By combining pharmacological, molecular, and lipidomic approaches, we demonstrate that the ABC-DLBCL subtype in particular displays a phenotype suggestive of high ROS burden and adaptive lipid targeted anti-oxidant defense characterized by high expression of core ferroptosis regulators, including FSP1, GCH1, and a MUFA enriched lipidome. In fact, elevated levels of MUFAs have been shown to render cells more resistant to ferroptosis [40] and, thereby, lipidome rewiring might represent a mechanism of cellular adaptation and anti-oxidant defense. Despite this, inhibition of GPX4 activity in DLBCL revealed that there is a higher dependence upon this defense pathway than in normal B-cells, underscoring the potential for selective tumor targeting.

Mechanistically, our work provides novel insight into redox regulation dependence of DLBCL. We show that ABC-DLBCL cells, in particular, exist in a state of heightened oxidative stress and rely on an intact antioxidant network for survival. While the PUFA content of DLBCL membranes was lower than in normal B-cells, the persistent oxidative burden and elevated expression of redox-regulatory genes appear to render these cells acutely dependent on GPX4 for lipid peroxide detoxification. This redox imbalance provides a biochemical basis for ferroptosis sensitivity and suggests that DLBCL cells are functionally addicted to ferroptosis-suppressive pathways.

Another key discovery of our study is that ibrutinib, a clinically approved BTK inhibitor, primes DLBCL cells for ferroptosis by scavenging GSH and downregulating GPX4 protein levels. Importantly, we demonstrate that ibrutinib acts through multiple noncanonical mechanisms, including chemical scavenging of GSH. Of note, GSH conjugation of ibrutinib was previously observed in human plasma [41] and seems to form part of the normal metabolic cycle of ibrutinib in patients. Here we find that this activity of ibrutinib forms part of the basis for ferroptosis sensitization. In addition, we find that broad inhibition of protein translation results in downregulated GPX4 protein levels translating into a lowered threshold for ferroptotic cell death. The ability of ibrutinib to suppress translation of short-lived proteins, including GPX4, cFLIP, and decrease of p-P70, which phosphorylates and activates the small ribosomal subunit, implicates a more global impairment of redox and survival buffering systems. This multifaceted disruption sensitizes DLBCL cells to even low concentrations of GPX4 inhibitors, leading to additive cell death that is fully reverted by the lipophilic radical-trapping agent ferrostatin-1. These findings redefine ibrutinib not just as a targeted kinase inhibitor but as a functional ferroptosis sensitizer with implications for its efficacy in patients. Notably, acute kidney injury, a hallmark feature of full-body GPX4 deletion in mice [14], has been observed as adverse effect in ibrutinib-treated patients [42]. Therefore, it is possible that the herein discovered activity of ibrutinib in ferroptosis sensitization is linked with kidney injury in patients and caution will have to be taken when designing a dose-regime. Yet, our data using primary human B-cells suggest that such therapeutic windows can likely be found but will have to be evaluated also in renal cells.

Clinically, our work has several important implications. First, it offers a rationale for the combinatorial use of BTK inhibitors and ferroptosis inducers in patients with relapsed or refractory DLBCL, particularly those with NF-κB–addicted ABC subtypes. Second, the selective ferroptosis sensitivity of malignant but not normal B-cells may mitigate the dose-limiting toxicities associated with conventional cytotoxic agents like cisplatin. While GPX4 inhbitiors do not meet pharmacokinetics for clinical application yet, the clinically approved drug dimethyl fumarate was found to induce ferroptosis in DLBCL in concert with the BCL2 inhibitor venetoclax (ABT199) [22]. Yet, venetoclax resistance associated with lack of BCL2 expression has been observed in DLBCL [43] and, hence, using additive therapy employing non-apoptotic mechanisms might result in more durable responses in these cases. Notably, ibrutinib is also part of the ViPOR regime (venetoclax, ibrutinib, prednisone, obinutuzumab, and lenalidomide), which was associated with durable remission in subsets of refractory DLBCL [35]. It is therefore possible that a part of the ViPOR mechanism involves priming to ferroptosis.

Several aspects of our study merit further investigation. The precise mechanisms by which ibrutinib impairs translation remain to be fully elucidated and may involve off-target engagement of kinases involved in mTOR signaling or ER stress pathways. Future studies in primary patient samples and in vivo models are needed to confirm the translational potential of this approach and to evaluate the safety and pharmacodynamics of combined BTK–GPX4 targeting in clinical settings. In addition, while ibrutinib additivity stemmed from GSH and GPX4 targeting, alternative GSH-independent ferroptosis sensitizers such as FSP1 inhibition could provide complementary routes to therapeutic induction of ferroptosis. In fact, interesting recent work identified that inhibitors against Bromodomain Containing 4 (BRD4) downregulate FSP1 thereby sensitizing DLBCL cells to ferroptosis [39].

In conclusion, this study defines ferroptosis as a baseline for additive therapy in DLBCL and demonstrates that BTK inhibition with ibrutinib can be exploited to enhance ferroptotic cell death. By disrupting lipid-directed antioxidant defenses at multiple levels, ibrutinib acts as a dual-function agent that not only suppresses survival signaling but also dismantles redox resistance, rendering DLBCL cells more susceptible to a non-apoptotic mode of cell death. This work establishes a mechanistic framework for integrating ferroptosis-based strategies into the therapeutic arsenal for aggressive, treatment-refractory lymphomas.

## Material and methods

### Reagents

ML210 (6429, Tocris), RSL3 (S8155, Sellekchem), ibrutinib (S2680, Selleckchem), Ferrostatin-1 (Cay17729, Cayman Chemicals), zVAD (ALX-260-020, Enzo Life Sciences), Nec1s (S8641, Selleckchem), Trolox (Cay10011659, Cayman Chemicals). C11 BODIPY 581/591 (D3861) and Draq7 (D15106) were purchased from Thermofisher (Germany).

### Cell culture

Human DLBCL cells were kindly provided by B. Chapuy, MCL cell lines were kindly provided by R. Jachimowicz, CLL cell lines were kindly provided by P.H. Nguyen and ALL cell lines were provided by L. Frenzel. Mouse DLBCL cell lines derived from the MBC-mouse model [32] were kindly provided by HC Reinhardt, the BCL2 overexpression allele used in the MBC-mouse model was previously generated and provided by H. Kashkar. All cells were cultured in RPMI-1640 (GIBCO) supplemented with 10% FCS (F7524, Sigma-Aldrich) and 1% Penicillin/Streptomycin (P06-07100, PAN-Biotech). Cells were kept at 37 °C with 5% CO_2_ and tested for mycoplasma at regular intervals (mycoplasma barcodes, Eurofins Genomics).

### Preparation of protein lysates

Cells were harvested by centrifugation at 1200 rpm for 5 min and washed once with PBS. After another centrifugation step the pellet was lysed in DISC lysis buffer (20 mM Tris, 150 mM NaCl, 2 mM EDTA, 10% Glycerin, 1% Triton-×-100, pH 7.4), supplemented with phosphatase (04906837001, Sigma-Aldrich) and protease (5056489001, Sigma-Aldrich) inhibitors on ice for minimum 1 h or overnight at −80 °C. Lysed cells were centrifuged at 14,000 rpm for 15 min at 4 °C. The supernatant containing the whole protein fraction was then transferred into a new reaction tube. Relative protein concentration was quantified by Bradford assay (5000113, 5000114, 5000115, BioRad) in Spark^®^ Multimode Microplate Reader (TECAN). 200 mM DTT (R0861, Thermo Scientific) containing NuPAGE LDS Sample Buffer (NP0008, Thermo Fisher Scientific) was added to the lysates and samples were boiled at 95 °C for 10 min.

### Western blot

Gel electrophoresis of proteins was performed using the Mini-PROTEAN® Tetra Cell System (Biorad). To resolve the proteins, sodium dodecyl sulfate polyacrylamide gel electrophoresis (SDS-PAGE) was run using Criterion TGX Stain-Free Precast Gels (BioRad, 5678085). Proteins were separated via gel electrophoresis at 200 V for 30 min in 1× Tris-Glycine SDS Running buffer. Precision Plus Protein™ Dual Color Standard (1610374, Bio-Rad) was included for size comparison. Proteins were transferred to a 0.2 mm nitrocellulose membrane (BioRad, 1704159) via trans-blot turbo transfer system (1704150, Bio-Rad). Membranes were blocked in 5% BSA (A8022, Sigma-Aldrich) in 1% v/v Tween20 TBS (blocking buffer) for 1 h at room temperature. Membranes were then incubated in primary antibodies diluted in blocking buffer overnight at room temperature. After secondary incubation, membranes were washed and the signal was detected using Enhanced Chemiluminescence reagents (PerkinElmer, NEL105001EA). The signal was visualized by exposing the membranes to X-ray films (Valmex, 807947) or with the FUSION Solo S system and software. Uncut blot images are provided in the Supplementary Material.

### Antibodies

Primary antibody species specific light chain secondary antibodies conjugate to HRP (JIM-115-035-174, JIM-112-035-175, JIM-211-032-171, Jackson ImmunoResearch) were used for signal detection on X-Ray film via a chemiluminescent substrate (NEL105001EA, PerkinElmer). The following antibodies were used GPX4 (Abcam, ab125066), FSP1 (Proteintech, 68049-1-Ig), GCH1 (Abnova, H00002643-M01), ACSL4 (Santa Cruz, sc-271800), LPCAT3 (abcam, ab232958), CD71 (Santa Cruz, sc-65882), p-P65 (CST, 3033S), IkBa (CST, 4812), total ubiquitin (Millipore, 07-375), GPX8 (ABGENT, AP16753b), GPX2 (Invitrogene, PA5-27150), TRXR1 (CST, 15140S), p-P70 (CST, #9206), cFLIP (CST, 56343), c-MYC (CST, 5605), MCL-1 (CST, 5453), xCT (abcam, ab37185), β-Actin (Sigma, A1978). For secondary antibodies α-rabbit IgG (211-032-171) and α-mouse IgG (115-035-174) were purchased from Jackson ImmunoResearch.

### Cell viability assays

Cells were seeded into 96-well plates (2 × 104 cells/well) and treated with respective drugs at indicated concentrations. Viability was measured after 48 h of treatment, if not stated otherwise, using a Spark® Multimode Microplate Reader (TECAN) utilizing CellTiter-Glo® Luminescent Cell Viability Assay (G7573, Promega), according to the manufacturers protocol.

### Cell death assays

Cells were seeded into 96-well plates (2 × 104 cells/well) and treated with the respective drugs indicated concentrations. Dead cells were stained by adding 0.25 µg/ml Propidium Iodide (Sigma-Aldrich; P4864) to all wells, followed by a 30 min incubation in the dark on ice before reading out their incorporations using a BD LSR Fortessa™ Cell Analyzer. Analysis was performed using the FlowJo^TM^ Software (V10.8.1).

### Cellular GSH quantification

To quantify concentrations of cellular GSH and GSSG, the GSH/GSSG Glo® Assay (Promega, V6611) was used according to the manufacturer’s instructions using 5 × 104 cells per well cells.

### MCB assay

For cell-free measurements 25 µM IBR was added to phenol-red free RPMI-1640 (+ 10% FCS, +1% P/S) in the presence or absence of 50 µM GSH. 50 µM MCB (69899, Sigma-Aldrich) was added, and the solution was incubated in the dark for 30 min on an orbital shaker. Fluorescence at Ex/Em: 394/490 nm was measured with a Spark® Multimode Microplate Reader (TECAN).

To measure the thiol content within U2932 cells 2 × 104 cells/well were treated with or 10 µM H_2_O_2_ (H1009, Sigma) for 2 h. 50 µM MCB was added for the last 30 min. Afterwards the plate was centrifuged and cells were washed and resuspended in phenol-red free medium, followed by cell lysis in an ultrasound bath. After a centrifugation step to spin down the cell debris 150 µL supernatant was transferred to a white-bottom 96-well plate and fluorescence was measured at Ex/Em: 394/490 nm with a Spark® Multimode Microplate Reader (TECAN).

### ROS quantification

For lipid ROS quantification, 2 × 104 cells/well were treated with 25 µM IBR, 50 nM ML21,0 either individually or combined in the presence or absence of 1 µM Fer-1 for 2.5 h. 5 µM BODIPY C11 per well was added for the last 30 min. Cells were then washed and green fluorescence was readout counting 10,000 cells by flow cytometry using a BD LSRFortessa™ Cell Analyzer. Analysis was performed using the FlowJo^TM^ Software (V10.8.1).

General ROS was stained using H2DCFDA staining. 2 × 104 cells/well U2932 cells were treated with 25 µM IBR, 50 nM ML210 individually or combined in the presence or absence of 1 µM Fer-1 or 50 µM Trolox for 2 h. Fot the last 30 min 20 µM H2DCFDA was added per well. Then, cells were washed and green fluorescence was readout counting 10,000 cells by flow cytometry using a BD LSRFortessa™ Cell Analyzer. Analysis was performed using the FlowJo^TM^ Software (V10.8.1).

### PBMC and B-cell isolation

Human PBMCs were isolated from buffy coats from healthy donors under an existing ethics approval at the University Hospital Cologne (21-1532) following written informed consent. We have complied with all relevant ethical regulations pertaining to the use of human patient material. Isolation was performed using Lymphopure (#426202, BioLegend) and SepMate-50 (StemCell Technologies, 353713) according to the manufacturer’s protocol. Subsequent human B-cell isolation was performed by a MACS depletion system using the human CD43 MicroBeads (Miltenyi Biotec; 130-091-333) and positive selection columns (Miltenyi, 130-042-401) according to the manufacturers protocol. B-cells were cultured in RPMI1640 supplemented with 10% FCS and 1% Penicillin/Streptomycin.

Murine splenic B-cells were isolated from spleens. The spleens were mashed through a 70 µM cell strainer and centrifuged at 350 × *g* for 5 min. Red blood cell lysis was performed using 5 ml ACK lysis buffer and incubated at room temperature for 3 min. Then, a washing step using PBS supplemented with 2% FCS was performed. Cell viability was measured using a Countess^TM^ 3 Automated Cell Chamber (ThermoFisher). Afterwards, B-cells were isolated from splenocytes using the mouse CD43 (Ly-48) MicroBeads (Miltenyi Biotec; 130-049-801) and LD depletion columns (Miltenyi, 130-042-901) according to the manufacturers protocol.

### Isolation of RNA and quantitative RT-PCR

Total RNA was isolated from cells after treatment with 25 µM IBR, 50 nM ML210 for 48 h using the NucleoSpin RNA Kit (740.955.250, Machery Nagel) according to the manufacturer’s protocol. To determine the RNA concentration a Nanodrop 8000 spectrophotometer was used. cDNA synthesis from the isolated RNA was reverse transcribed using the LunaScript RT SuperMix Kit according to the manufacturer’s protocol. Real-time qPCR was performed in quadruplets on the Quant Studio 5 qRT-PCR machine. Relative expression of gene transcripts was analysed via the 2-ΔΔCT method to the reference gene 60S ribosomal protein L13a (RFL13A).

The following primer sequences were used:

RPL13A forward GCCATCGTGGCTAAACAGGTA

RPL13A forward GTTGGTGTTCATCCGCTTGC

GPX4 forward GAGGCAAGACCGAAGTAAACTAC

GPX4 reverse CCGAACTGGTTACACGGGAA

### Bioinformatic analysis of mutational signatures

Mutational signatures were de novo inferred in the ICGC/PCAWG MALY-DE cohort (*n* = 107, EGAD00001002123) from the European genome-phenome archive (EGA), which includes samples from DLBCL, FL, and Burkitt lymphoma. Using SigProfilerExtractor (v1.2.1), we identified six distinct mutational signatures. One of these, SBS96C, showed a high cosine similarity (0.85) to COSMIC signature SBS18. Subtype information was available for 57 of the samples [44], enabling us to plot signature activity accordingly.

### Synergy calculations

Synergy Calculations were performed using SynergyFinder 3.0 [45] analyzing the BLISS score.

### Quantification and statistical analysis

Statistical analysis was performed using GraphPad software (GraphPad Software Inc.). Two-tailed *t*-tests were used to compare two conditions, and two-way ANOVA was used to compare multiple samples. For mutational signatures a wilcoxon rank sum test was performed. All measurements were performed at least three times, and results are presented as mean ± standard deviation. Western Blot quantification was performed with ImageJ 1.54 × *g*.

### Lipidomics

Glycerophospholipids (PC and PE, including ether-linked species) in cells were analyzed by Nano-Electrospray Ionization Tandem Mass Spectrometry (Nano-ESI-MS/MS) with direct infusion of the lipid extract (*Shotgun Lipidomics*): Approximately 2 × 106 cells were homogenized in 250 µl of Milli-Q water using the Precellys 24 Homogenisator (Peqlab) at 6500 rpm for 30 s.

To 100 µl of the homogenate 400 µl of Milli-Q water, 1.875 ml of methanol/chloroform 2:1 (v/v) and internal standards (63 pmol PC 17:0-20:4 and 69 pmol PE 17:0-20:4, Avanti Polar Lipids) were added. Lipid extraction and Nano-ESI-MS/MS analysis were performed as previously described [46]. Endogenous glycerophospolipids were quantified by referring their peak areas to those of the internal standards. The calculated glycerophospholipid amounts were normalized to the cell count in the cell homogenate.

## Supplementary information

Supplementary Data

Original Data file

## Data Availability

All original data are available from the corresponding authors upon reasonable request.
