# Supplementary material for: Redox destabilization by ibrutinib promotes ferroptosis in diffuse large B-cell lymphoma (DLBCL)
Source: Cell Death Discov. 2025 Oct 31;11:495. doi: 10.1038/s41420-025-02826-w (PMC12578790; doi:10.1038/s41420-025-02826-w)
Supplement: Supplementary file 1 — Supplementary Data [file 41420_2025_2826_MOESM1_ESM.pdf]

Supplementary Data File

Redox destabilization by ibrutinib promotes ferroptosis in diffuse large B-cell lymphoma (DLBCL)

Anuschka Langpape[1,2,3], Debora Bonasera[3,4,5], Jenny Stroh[1,2], Moritz Reese[1,2], Maria Cartolano[1], Gianmaria Liccardi[2,3,6]* and Silvia von Karstedt[1,2,6] *

[1]University of Cologne, Faculty of Medicine and University Hospital Cologne, Department of Translational Genomics, Cologne, Germany.

[2]CECAD Cluster of Excellence, Faculty of Medicine and University Hospital Cologne, Cologne, Germany.

[3] Genome instability, inflammation and cell death laboratory, Institute of Biochemistry I, Centre for Biochemistry, Faculty of Medicine, University of Cologne, 50931 Cologne, Germany.

[4]Cell death, inflammation and immunity laboratory, Institute of Biochemistry I, Centre for Biochemistry, Faculty of Medicine, University of Cologne, 50931 Cologne, Germany.

[5]Cell death, inflammation and immunity laboratory, CECAD Cluster of Excellence, University of Cologne, 50931 Cologne, Germany.

[6]Center for Molecular Medicine Cologne, Faculty of Medicine and University Hospital Cologne, Cologne, Germany.

*Co-Last and Co-corresponding authors G.L. and S.v.K.: Gianmaria.liccardi@uk-koeln.de; s.vonkarstedt@uni-koeln.de

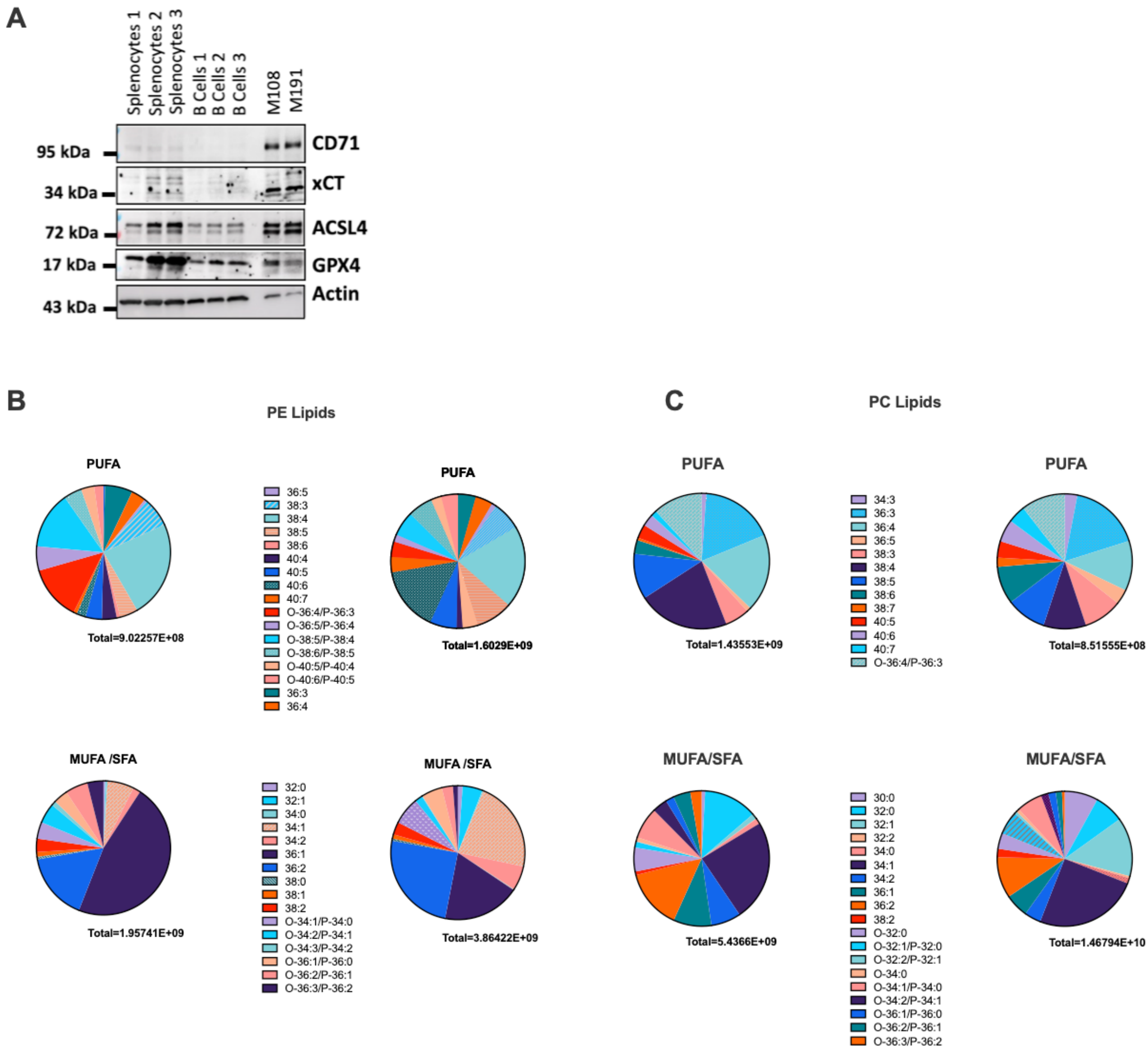

Suppl Fig.1 DLBCL cells lines upregulated mono-unsaturated fatty acids to prevent Ferroptosis activation. a Representative western blot of ferroptosis pathway component expression in murine lymphoma cell lines compared to WT Splenocytes and matched B Cells. **b** Phosphatidylethanolamine (PE) lipid species and **c** Phosphatidylcholine(PC) lipid composition measured by lipidomic analysis of n=3 ABC-DLBCL cell lines (HBL1, TMD8, U2932) compared to n=3 healthy donor B Cells isolated from PBMCs from buffy coats. PUFA = Polyunsaturated Fatty Acids. MUFA = Monounsaturated Fatty Acids.

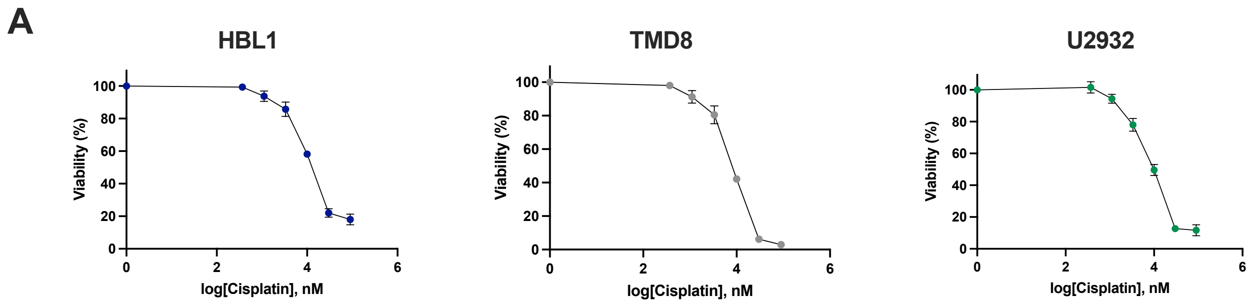

Suppl. Fig. 2 DLBCL are sensitive to cisplatin treatment. a Viability measurements of human ABC-DLBCL cell lines by CellTiter-Glo® following 48 hours treatment with increasing cisplatin concentrations. Data are mean ± SD for each cell line of n=3 independent experiments.

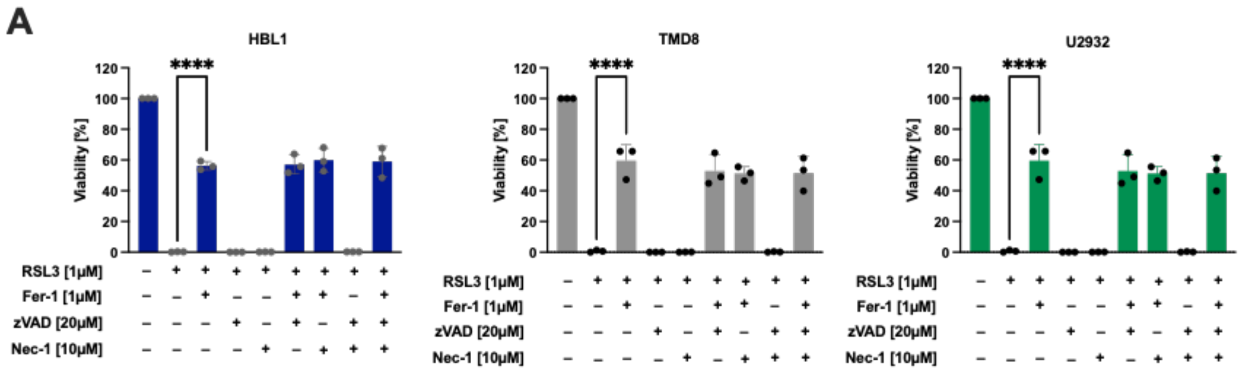

Suppl. Fig. 3 DLBCL are sensitive to RSL3 induced ferroptosis. a Viability measurements of human ABC-DLBCL cell lines by CellTiter-Glo® following 48 hours treatment indicated concentrations of RSL3 in combination with Fer-1 and/or zVAD and/or Nec-1s. Data are mean ± SD for each cell line of n=3 independent experiments. *=p value 0.05, **=p value 0.01, ***= p value 0.001, ****= p value 0.0001.

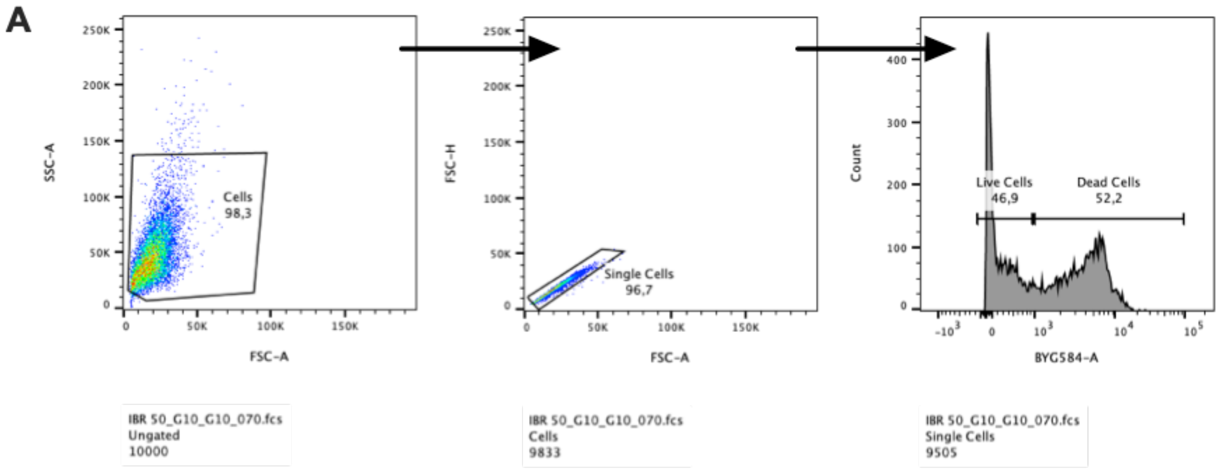

Suppl. Fig. 4 Gating strategy of Flow cytometric analysis. a Gating Strategy for flow cytometric analysis.

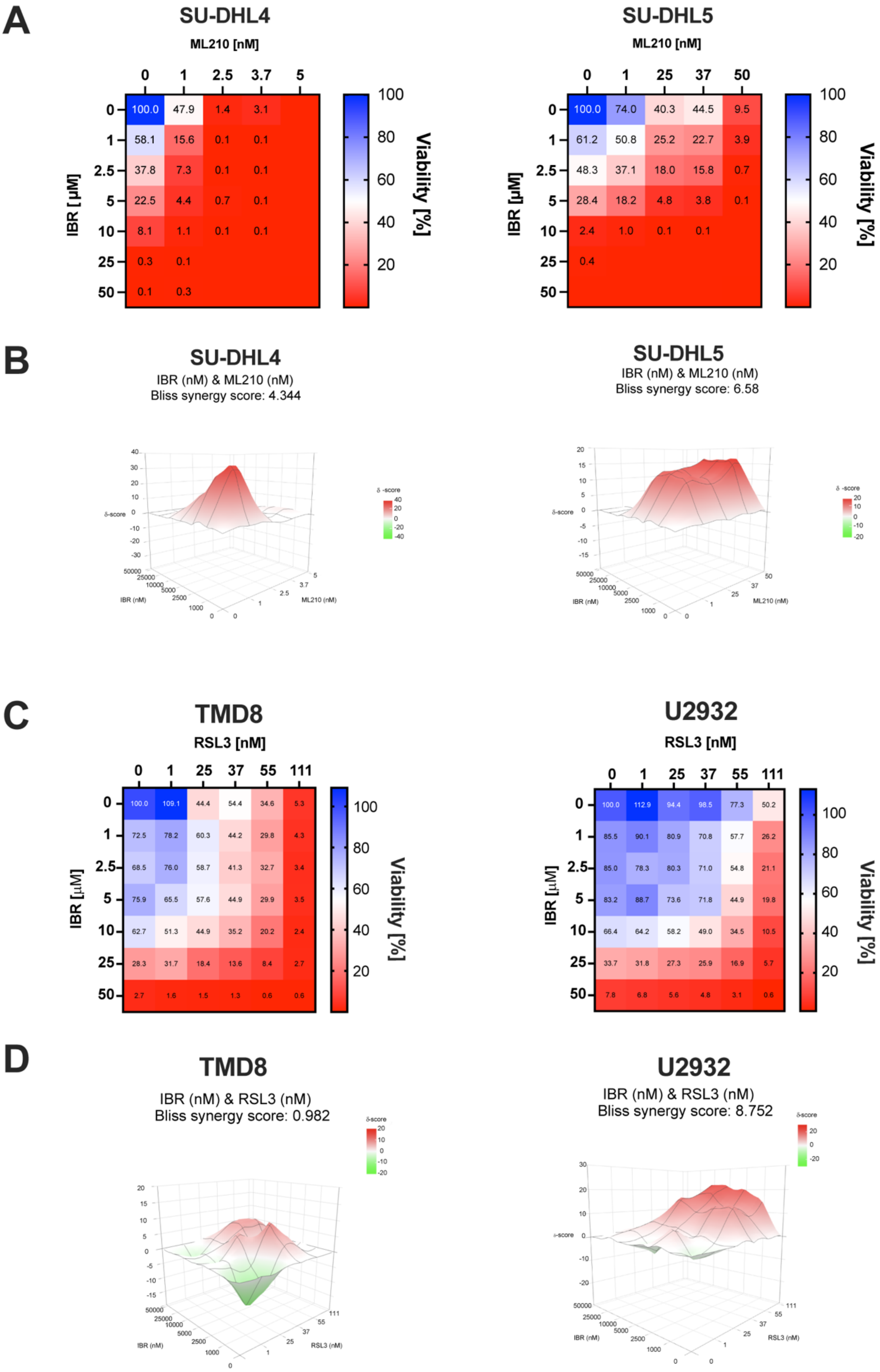
A
SU-DHL4
ML210 [nM]
0 1 2.5 3.7 5
IBR [μM]
0 1 2.5 5 10 25 50
100.0 47.9 1.4 3.1
58.1 15.6 0.1 0.1
37.8 7.3 0.1 0.1
22.5 4.4 0.7 0.1
8.1 1.1 0.1 0.1
0.3 0.1
0.1 0.3
Viability [%]
100 80 60 40 20
SU-DHL5
ML210 [nM]
0 1 25 37 50
IBR [μM]
100.0 74.0 40.3 44.5 9.5
61.2 50.8 25.2 22.7 3.9
48.3 37.1 18.0 15.8 0.7
28.4 18.2 4.8 3.8 0.1
2.4 1.0 0.1 0.1
0.4
Viability [%]
B
SU-DHL4
IBR (nM) & ML210 (nM)
Bliss synergy score: 4.344
SU-DHL5
IBR (nM) & ML210 (nM)
Bliss synergy score: 6.58
C
TMD8
RSL3 [nM]
0 1 25 37 55 111
IBR [μM]
100.0 109.1 44.4 54.4 34.6 5.3
72.5 78.2 60.3 44.2 29.8 4.3
68.5 76.0 58.7 41.3 32.7 3.4
75.9 65.5 57.6 44.9 29.9 3.5
62.7 51.3 44.9 35.2 20.2 2.4
28.3 31.7 18.4 13.6 8.4 2.7
2.7 1.6 1.5 1.3 0.6 0.6
Viability [%]
U2932
RSL3 [nM]
0 1 25 37 55 111
IBR [μM]
100.0 112.9 94.4 98.5 77.3 50.2
85.5 90.1 80.9 70.8 57.7 26.2
85.0 78.3 80.3 71.0 54.8 21.1
83.2 88.7 73.6 71.8 44.9 19.8
66.4 64.2 58.2 49.0 34.5 10.5
33.7 31.8 27.3 25.9 16.9 5.7
7.8 6.8 5.6 4.8 3.1 0.6
Viability [%]
D
TMD8
IBR (nM) & RSL3 (nM)
Bliss synergy score: 0.982
U2932
IBR (nM) & RSL3 (nM)
Bliss synergy score: 8.752

Suppl. Fig. 5 GPX4 inhibition treatement has an additive effect in combination with Ibrutinib. **a-d** Cell viability of the indicated human **a** GCB-DLBCL **c** ABC-DLBCL cell lines following 48 hours treatment with increasing concentrations of **a** ML210 or **c** RSL3 in combination with IBR as determined by CellTiter-Glo®. Heatmap color code indicates viability levels of each sample. **b,d** BLISS synergy scores were determined using https://synergyfinder.fimm.fi.

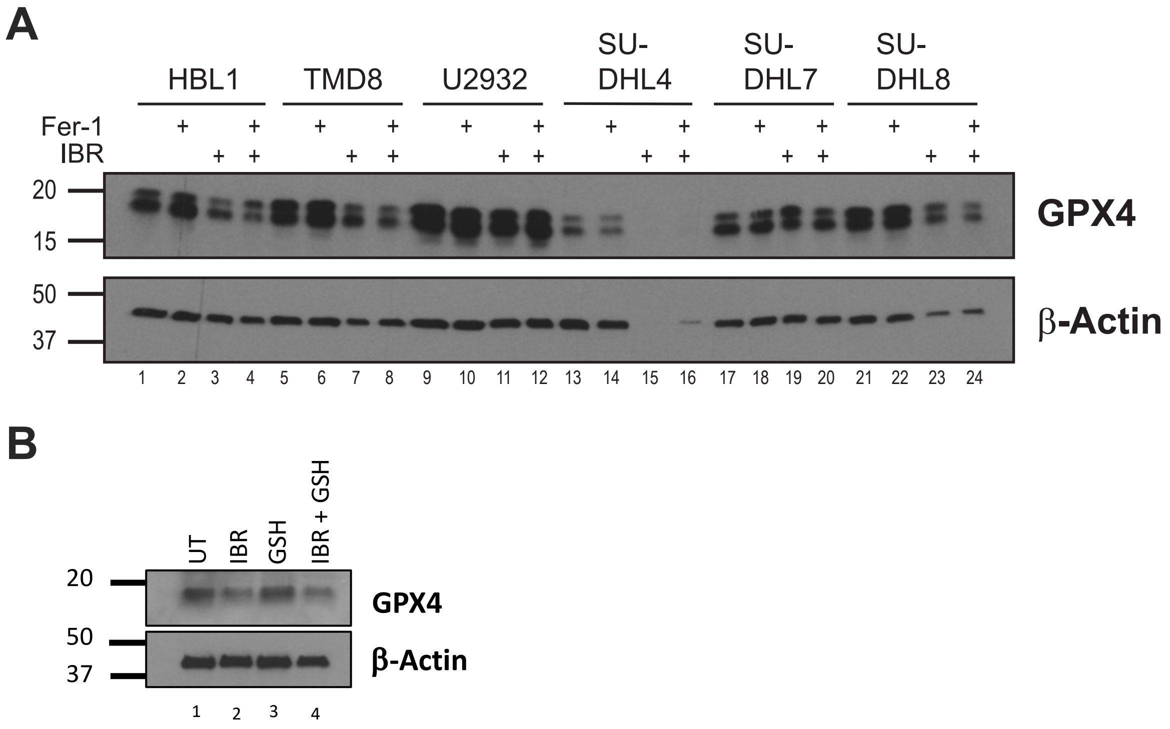

Suppl. Fig. 6 Ibrutininb downregulates GPX4 protein levels independently of Glutathione. **a** Western blot analysis of indicated DLBCL cell lines following 48h of treatment with IBR [10 µM] and/or Fer-1 [1 µM]. **b** Western blot analysis of U2932 cells treated with IBR [25 µM] and/or GSH [2 mM].

A

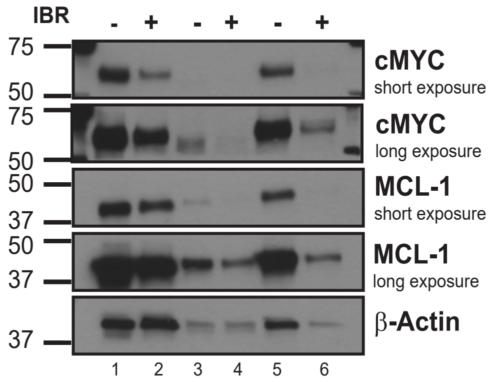

Suppl. Fig. 7 Ibrutininb downregulates translation of short-lived proteins. a Replicates of western blot of U2932 cells untreated or treated with IBR [25 µM] in the presence of Fer-1 [1 µM] for 24h.
