# Supplementary figures and images for: Redox destabilization by ibrutinib promotes ferroptosis in diffuse large B-cell lymphoma (DLBCL)
Source: Cell Death Discov. 2025 Oct 31;11:495. doi: 10.1038/s41420-025-02826-w (PMC12578790; doi:10.1038/s41420-025-02826-w)

28/01-25  
30/01-25

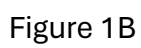

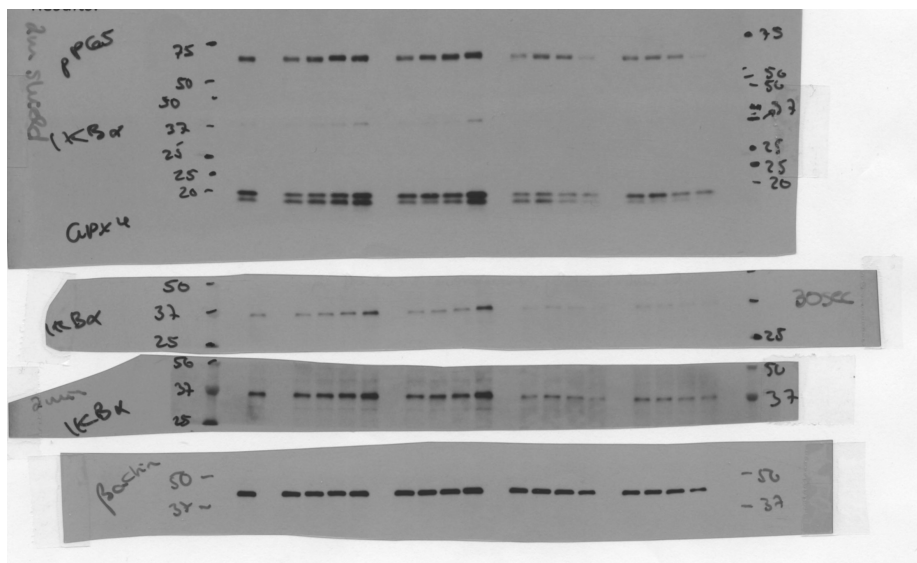

Figure 4E

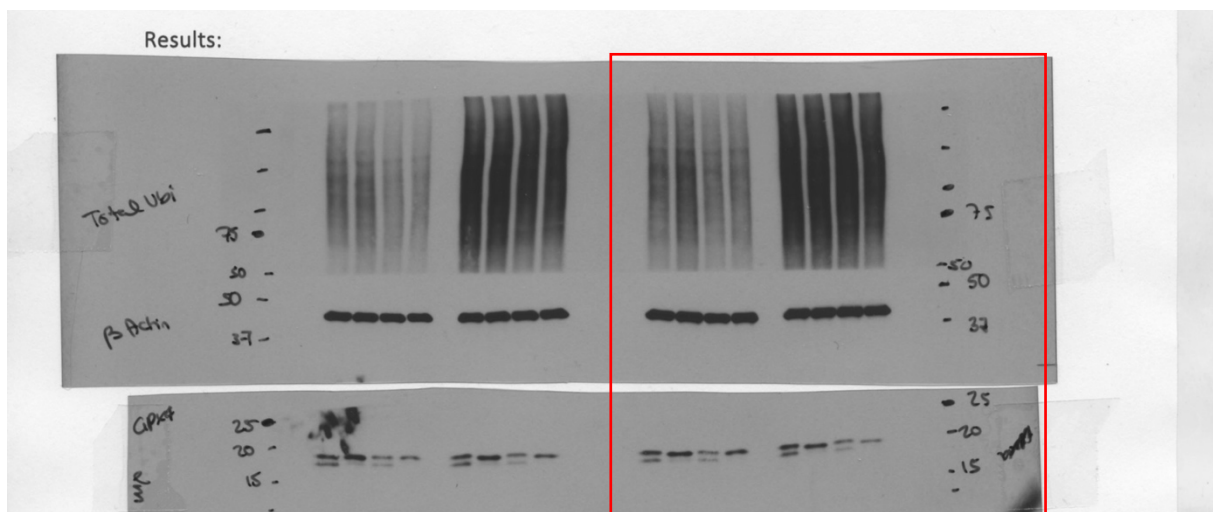

Figure 4F

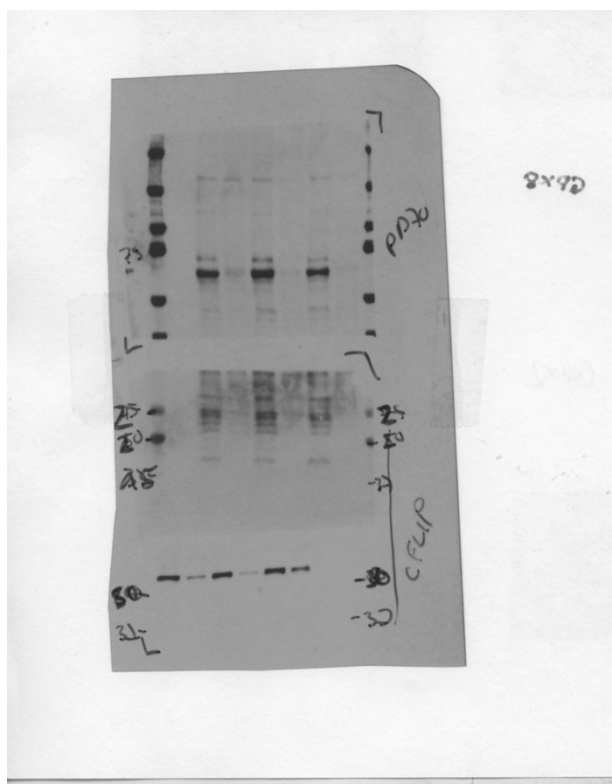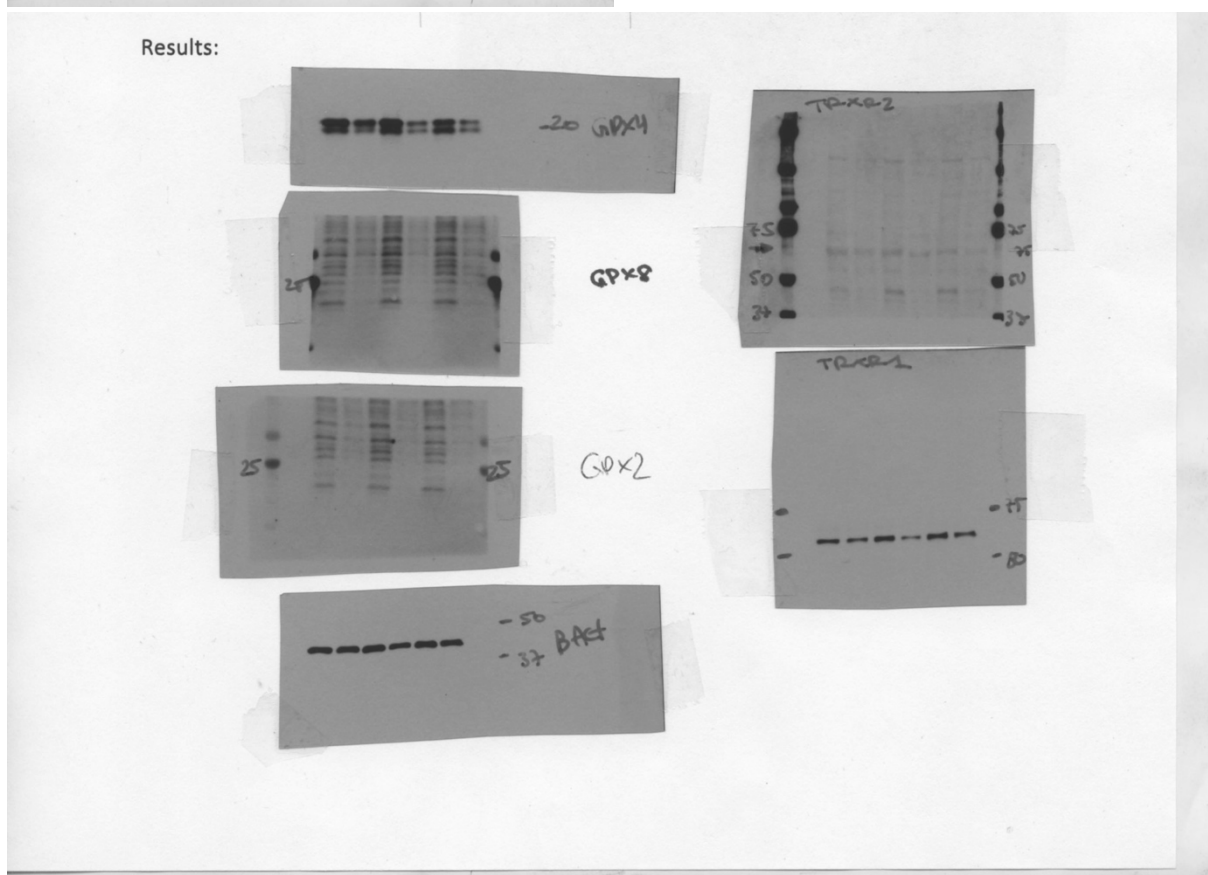

Figure 41

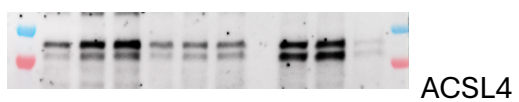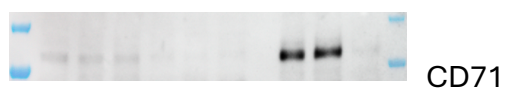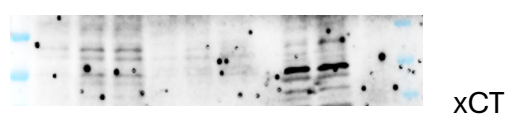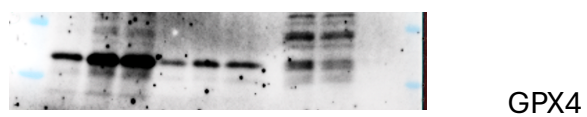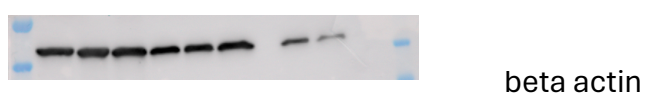

Suppl. Fig. 1A

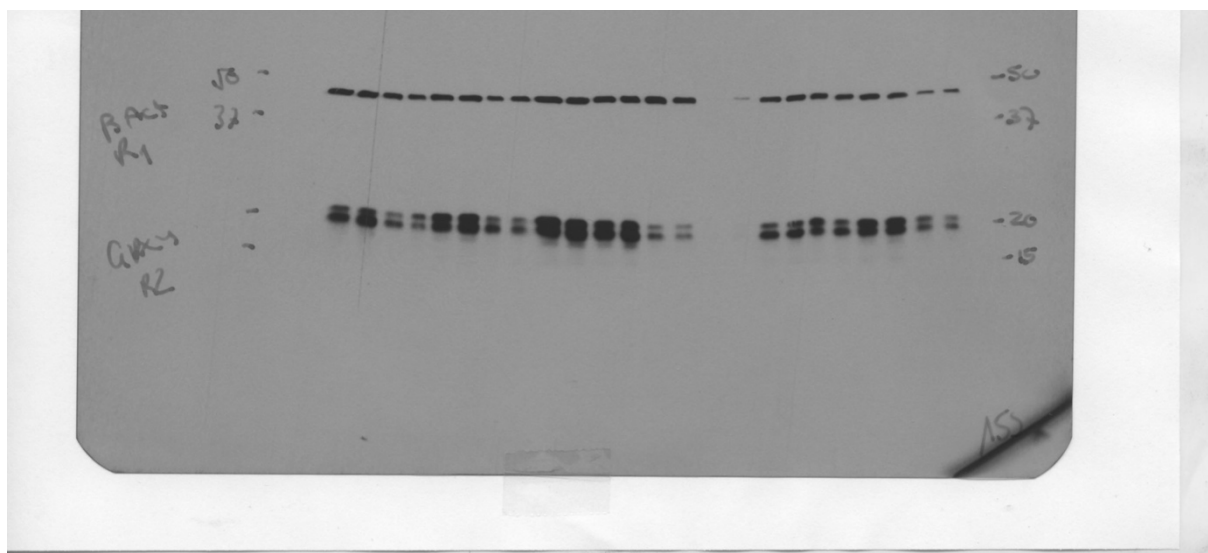

Suppl. Fig. 4A

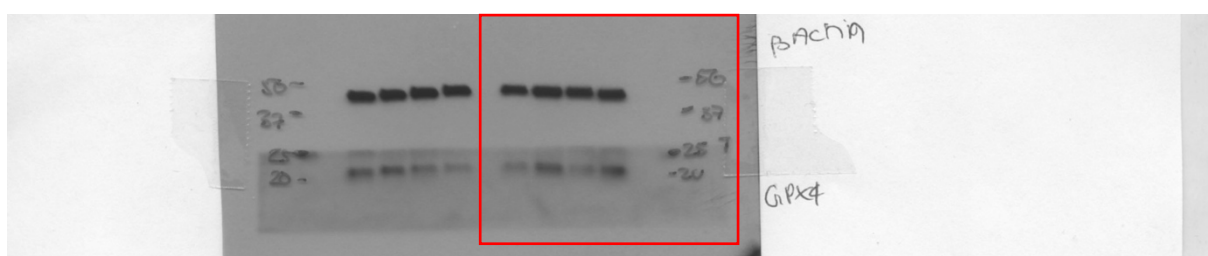

Suppl. Fig. 4B

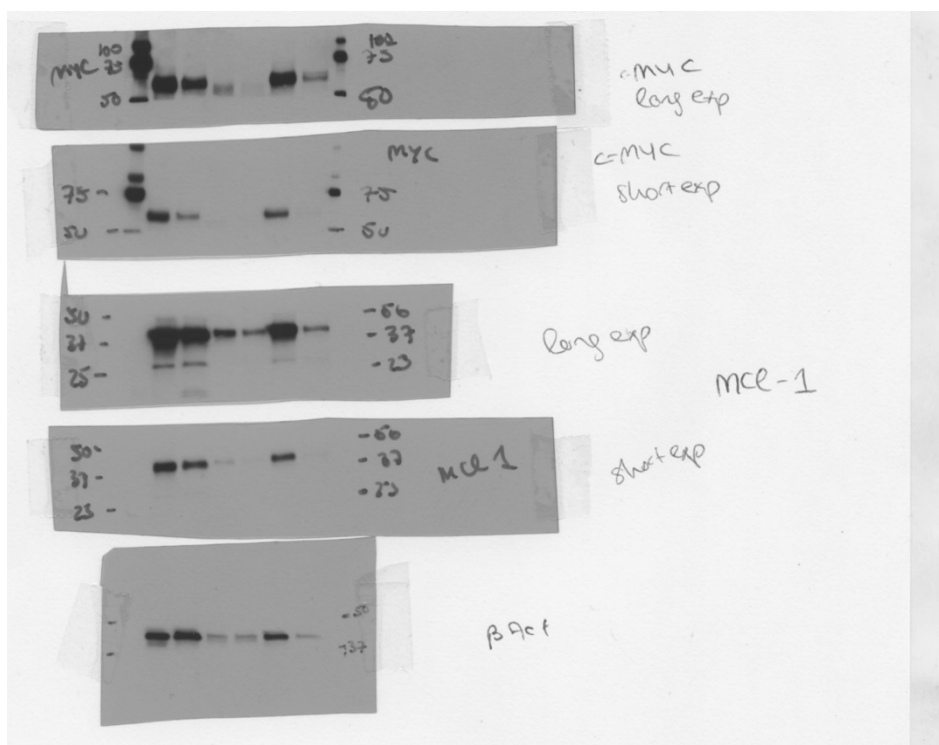

Suppl. Fig. 7A

Supplement: Supplementary file 2 — Original Data file [file 41420_2025_2826_MOESM2_ESM.pdf]
